# Scale-up of Emulsion Polymerisation up to 100 L and with a Polymer Content of up to 67 wt%, Monitored by Photon Density Wave Spectroscopy

**DOI:** 10.3390/polym14081574

**Published:** 2022-04-12

**Authors:** Laurence Isabelle Jacob, Werner Pauer

**Affiliations:** Institute for Technical and Macromolecular Chemistry, University of Hamburg, Bundesstraße 45, 20146 Hamburg, Germany; laurence.jacob@chemie.uni-hamburg.de

**Keywords:** scale up, inline analytics, photon density wave spectroscopy, emulsion polymerisation, mean particle size

## Abstract

The scale-up process of the high solid content (up to 67 wt%) emulsion polymerisation of vinyl acetate and Versa^®^10 from 1 L over 10 L to 100 L was investigated. An emulsion copolymerisation of vinyl acetate and neodecanoic acid vinyl ester in a molar ratio of 9:1 was carried out in a starved-fed semi-batch operation. As a radical source, a redox initiator system consisting of L-ascorbic acid, tert-butyl hydroperoxide and ammonium iron (III) sulphate was used. The process parameters, such as the required stirring speed and heat dissipation, were determined and adjusted beforehand via reaction calorimetry to ensure a successful scale-up without safety issues. In addition, the emulsion polymerisation was monitored inline by Raman (qualitative monomer accumulation), as well as Photon Density Wave spectroscopy (particle size and scattering coefficient) and temperature measurements. The data provided by Raman spectroscopy and temperature measurements revealed mixing difficulties due to an insufficient stirring rate, while the inline measurement with Photon Density Wave spectroscopy offered an insight into the development of the product properties. It proved to be reliable and precise throughout the entire scale-up process, especially compared to conventional offline methods, such as dynamic light scattering or sedimentation analysis by means of a disc centrifuge, both of which encountered issues when using higher polymer contents.

## 1. Introduction

Emulsion polymerisation is the most commonly used process for the production of water-borne latex polymers, and its importance in the industry keeps growing. It is a free radical polymerisation method carried out in a heterogenous reaction system, and commercially available polymer latex products usually contain around 40–55 wt% of solid content [1].

Before a polymer latex can be transferred from a laboratory scale into production, the process needs to be thoroughly examined, for example, regarding reproducibility or heat transfer, and then successfully scaled up to a larger set-up. The following work focuses on the scale-up process of the emulsion polymerisation of vinyl acetate and Versa^®^10 from a 1 L scale to a 10 L and 100 L scale while achieving a high solid content of over 60 wt%. Moreover, this article examines the possibility of monitoring the scale-up process by using an inline method to measure the particle size.

A commonly used monomer in emulsion polymerisation is vinyl acetate, which is not only relevant in the industry but has also proved to be interesting for researchers [2]. Emulsion polymerisation of vinyl acetate and Versa^®^10 with a high solid content of over 60 wt% was successfully performed in the past and showed an excellent agreement between offline and inline particle size measurement methods up to a solid content of 36 wt% [3]. However, agglomeration of particles at polymer content of 40–50 wt% created difficulties for further comparisons, which made a revision of the recipe necessary before considering upscaling. Pohn et al. developed a CFD model to simulate an upscaling from 1 L to 100 L and found it challenging to describe an emulsion polymerisation process in a 100 L stirred vessel with a turbine stirrer, leading to a laminar regime. They also came across the same issues regarding coagulation, resulting in a secondary population of larger particles [1]. To avoid encountering the same difficulties, this work focused on improving the recipe on the one hand and modifying the set-up on the other hand. As a laminar regime does not seem suitable for the upscaling of emulsion polymerisation at high polymer contents, the process described in this work shows a set-up involving an anchor stirrer, instead of a turbine stirrer, leading to a turbulent regime.

### 1.1. Scale-Up

The scale-up process remains one of the major challenges of chemical engineering. It allows the building of a bridge between an innovation that occurred on a laboratory scale and an actual invention that works in an industrial environment. Upscaling a process can often reveal difficulties that were not detected on a smaller scale. Building a pilot plant is, therefore, a crucial step before transferring a new product into production. However, due to the numerous factors that can influence the product properties and process development, there is no standard recipe for a scale-up process, and success often lies in the hands of experience, successful ideas, and many mistakes, which in the end, can lead to the desired outcome [4,5]. Nonetheless, guidelines exist that are designed to offer a starting point in the planning of such a project. Firstly, it is recommended to avoid cross-influences by keeping the set-up as identical as possible regarding the measurements of the reactor, the dosing units, dosing time, or molar ratio of the components. The size and width of the stirrer, as well as its speed, are also important factors of a scale-up. Modifying the flow or mixing characteristics can have a huge impact on the success of the polymerisation process. Besides these aspects, there is a list of rules commonly used and accepted that can be considered for the scale-up process [6]. The first one involves maintaining a constant stirring speed, regardless of the reactor size. This can, however, result in unobtainable power input and is therefore not adapted to all kinds of upscaling processes. The second and third rules indirectly apply to the size of the reactor, as they involve keeping either the stirring tip speed, which considers the size of the stirrer, or the circulation time constant, which depends on the diameter of the vessel. Another approach to the upscaling process involves keeping the Reynolds number constant. The Reynolds number is defined as a correlation between the density of the liquid, the viscosity, the stirring rate, and the diameter of the stirrer and is an important and dimensionless quantity used in fluid mechanics. It contributes to the description of the mixing quality and constitutes the main parameter of the scale-up process described in this work, as a turbulent regime was considered necessary. According to the fluid mechanics and when using an anchor stirrer, a turbulent regime is achieved when the Reynolds number of the stirring is higher than 10,000 [7]. Further rules, which can be applied when planning a scale-up process, are keeping the power input constant, or controlling the mean energy dissipation. Each of these rules aims at keeping a constant similarity of the process, regardless of the final scale. Past experience and published articles suggest employing a certain rule, depending on the requirements [8,9,10]. For example, according to Zhou and Kresta [11,12], for liquid–liquid dispersions, both energy dissipation and flow are the most important characteristics. Nonetheless, everyone agrees that a recipe that works for all processes does not exist.

### 1.2. Inline Monitoring

The successful upscaling of a process is not limited to the size of the set-up alone but also includes maintaining the desired product properties. This is why a thorough analysis of the dispersion during the scale-up process is crucial. Among other properties, the latex particle size and its distribution, for example, have an influence on the surface properties of the dried polymer film or, alternatively, can provide information about the kinetics of the reaction as well as the number of radicals per particle [13,14]. To obtain knowledge and control of the particle size, it is essential to use the right analytical methods [15,16]. The most common methods for particle size measurements are offline analysis, for example, dynamic light scattering (DLS) or disc centrifuge (DC). Offline analysis has many disadvantages, which can be traced back to the sampling and sample preparation, as most methods exclude high solid contents. However, a promising inline measurement method has gained interest in recent years, as it is able to monitor the particle size and scattering coefficient during the emulsion polymerisation process [17,18,19,20,21,22]. Photon density wave (PDW) spectroscopy has already been established in the fields of biochemistry and food chemistry but has lately proven to be a useful tool in the process control of emulsion polymerisation [3,23]. Studies on a laboratory scale showed a successful comparison of particle size measurements between PDW spectroscopy and common offline measurements methods, such as DLS [24] and sedimentation analysis by means of a DC [3]. Furthermore, Schlappa et al. [23] showed that the inline measurement of the reduced scattering coefficient could be useful for the real-time detection of modifications occurring in the process. For example, gelation could be recognised instantly, and appropriate countermeasures could be initiated immediately in order to save the product. This could also prove to be useful for a scale-up process.

PDW spectroscopy is an inline Process Analysis Technology (PAT) that can be used to monitor the reaction progress by measuring the optical properties of the dispersion, i.e., the absorption coefficient and the reduced scattering coefficient [17,18,19,20,22]. The measurement method uses the correlation between the reduced scattering coefficient and the particle size to determine the latter in real time in the dispersion by measuring the reduced scattering coefficient [22,25,26,27,28].

## 2. Materials and Methods

All chemicals were used directly without further purification. Each component of the reaction was flushed with nitrogen for at least 40 min, and the nitrogen flow inside the reactor was maintained during the whole process. The gas flow was fed into the reactor through a metal pipe with a diameter of 3 mm that was immersed into the initial charge. The amount of water evacuated by the continuous nitrogen flow was not taken into account for the 1 L process, as it was determined to be less than 1%. However, the amount of water evacuated during the 10 L and 100 L processes was calculated theoretically and confirmed by test runs. The amount of water lost at the end of the reaction was considered when determining the amount of monomer needed to achieve the desired polymer content. The latter was also confirmed via solid content determination (see method descriptions below).

For safety reasons, the first emulsion polymerisations were performed in a 1.8 L RC1e reaction calorimeter (Mettler Toledo) with a double jacket steel reactor (HP60, Mettler Toledo) to determine the heat generation during the process. The vessel had a diameter of 10.3 cm and was sealed with a non-heated steel lid. An anchor stirrer with a span of 9 cm was used to stir the dispersion. 

**Figure 1:**
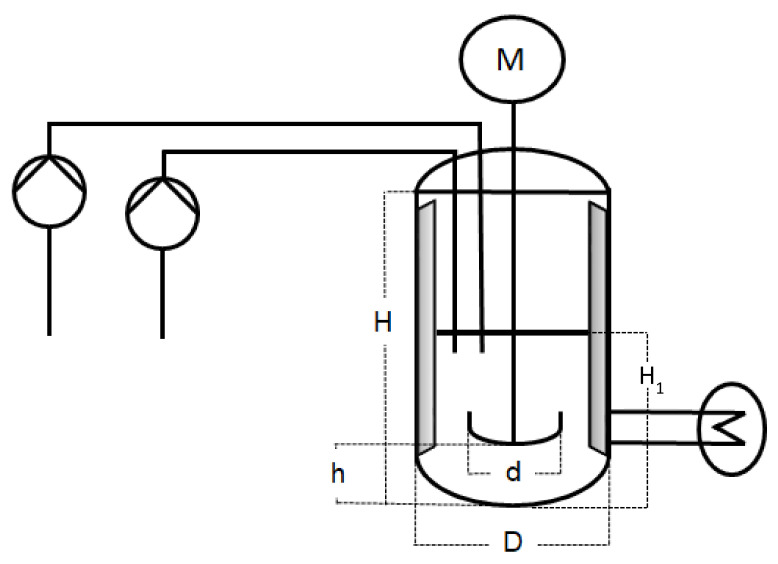
Schematic representation of the experimental set-up.

The scale-up emulsion polymerisations were carried out in three different reactors of, respectively, 1 L, 10 L, and 100 L. The 1 L reactor was a double-jacket glass vessel, whereas the 10 L and 100 L reactors were double-jacket reactors made of steel. Each reactor had a dish-like bottom with the sample outlet in the middle. For the sake of comparability, all three set-ups were kept as identical as possible. In scale-up processes, it is common to keep a constant height to diameter ratio. The three reactors used for the experiments described in this work were not customised and therefore cannot fully meet this criterion. However, while H usually refers to the total height of the vessel, when considering the filling height at the beginning of the reaction, the H_1_/D (cf. Figure 1 and Table 1) ratio is 0.25 in all three reactor sizes. The exact measurements are summarised in Table 1 and represented in Figure 1 for a better understanding.

In order to achieve a better mixing of the components at all stages of the reaction, a stainless-steel anchor stirrer was used in combination with two baffles [29]. To avoid forming a water swirl at the beginning of the reaction, when only water and emulsifier were present, baffles were inserted into the reactor to interrupt the flow and to enable a better mixing of the components. The width of the baffles amounted to 1/12 of the reactor diameter and they were placed at a distance of 1/72 from the tank wall [30,31]. The anchor stirrer had a width of 0.87.D of the respective vessel, and 0.95.D when deducting the baffles [32,33]. The stirring rate is summarised in Table 2 and determined by the Reynolds number (cf. Table 3).

The emulsion polymerisations were carried out as a semi-batch, closed-loop, controlled and starved–fed process at 1 bar. The initial charge contained demineralised water with a conductivity of 0.8 uS.cm^−1^ (see Table 4 for exact amount), 0.20 g.L^−1^ ammonium iron (III) sulphate (Merck KGaA) as a catalyst, and 50.4 g.L^−1^ Mowiol 4–88 (Sigma-Aldrich, St. Louis, MO, USA) as an emulsifier, resulting in a final emulsifier fraction of 2.44 wt% based on the total amount of monomer.

All experiments, including the test runs in the reaction calorimeter, were performed identically as described hereafter. The initial charge and feeding rates were summarised in Table 4.

The temperature of the reaction solution was brought to 60 °C within, respectively, 30, 60, and 120 min while flushing its content with nitrogen for at least 40 min before starting the reaction. The temperature of the 1 L, 10 L and 100 L reactor was regulated, respectively, by a Julabo Cryo Compact F30-C thermostat, a Huber Unistat Tango Thermostat, and a Huber Unistat 405wl Thermostat with constant jacket temperature.

Two dosing units were used; the first contained the monomer mixture, consisting of vinyl acetate and neodecanoic acid vinyl ester (Versa^®^10, Wacker Chemie AG, Burghausen, Germany) in a molar ratio of 9:1. The feeding rate can be found in Table 4.

The second dosing unit contained a premixed 3.4 wt% solution of, respectively, the reducing and the oxidising agents, L-ascorbic acid (AsAc, Sigma-Aldrich, St. Louis, MO, USA) and tert-butyl hydroperoxide (tBHP, Sigma-Aldrich, St. Louis, MO, USA), cf. Table 5. The feeding rate can be found in Table 4.

The dosing occurred through PTFE hoses with an inner diameter of 3 mm. The end of each hose was immersed into the reaction solution so that the dosing substance would be immediately stirred into the reactor content. For the 1 L reactor, the oxidising and reducing agents were fed with a syringe pump from kdScientific and using polyethylene syringes from Henke-Sass, Wolf GmbH. The monomer feed for the 1 L reactor and both feeds for the 10 L and 100 L reactor were performed with a precision SyrDos syringe pump from Hitech Zang using 2.5 mL syringes.

The feeding of the reducing and oxidising agents was started first. After 5 min, the dosing of the monomer mixture was started and stopped after achieving a polymer content of 63–67 wt% based on the total mass. The polymer content was checked via microwave analyser. The feeding of the reducing and oxidising agents was continued for another 10 min before they were stopped. The catalyst was added to the reaction solution diluted in 1 mL demineralised water by using a polyethylene syringe from Henke-Sass, Wolf GmbH, just before starting the monomer dosing unit. The total dosing time was 7.33 h.

When taking a sample from the 1 L and 10 L or 100 L reactor, the reaction was stopped with, respectively, 1 mL and 10 mL of a 1.7 m% solution of hydroquinone (Sigma-Aldrich, St. Louis, MO, USA). A sample was taken at, respectively, 10, 20, 30, 40, 50, 60, and 63 wt% polymer content to be able to determine the course of the particle size as a function of the polymer content and to verify a complete conversion at all times.

Each emulsion polymerisation considered in the following work was replicated three times to ensure a reproducible outcome.

### 2.1. Determination of Yield

Samples were taken through the outlet at the bottom of the reactor (after discarding enough dispersion to compensate for the dead volume of the outlet) and measured by gas chromatography (GC) as well as by microwave analyser in order to determine the yield of the reaction. In addition to the offline determination methods, inline monitoring of the monomer amount was performed by Raman spectroscopy throughout the entire reaction.

#### 2.1.1. Gas Chromatography (GC)

The samples were measured with an Agilent 7820A using hydrogen as a carrier gas (column: CP-Sil 5CB fused silica, 30 m, 1.0 µm, detector: FID, injector temperature: 200 °C, detector temperature: 250 °C, sample volume: 0.4 μL).

Then, 500 mg of each sample was withdrawn with a 100–1000 µL Eppendorf research micropipette, weighed, and then dissolved in 5 mL of *N*,*N*-dimethylacetamide. Finally, 70 mg of toluol was added as an internal standard and also weighed. Once fully prepared and dissolved, 1.5 mL of the solution was transferred into an amber glass vial and sealed with a PTFE/silicone septum and measured.

#### 2.1.2. Microwave Analyser

The samples were measured with a Smart System 5 device from CEM. The microwave analyser measured the total solid content of the sample. Approximately 3 g of sample was weighed, dried at a temperature up to 120 °C and then weighed again by the device, which then determined the weight difference. A CEM glass fibre sample pad was used as a sample carrier and tared beforehand. The drying process was temperature-controlled through microwave radiation. This method of analysis allows the determination of the total solid content of the sample. The polymer content can then be determined by subtracting all other solid components, such as Mowiol 4–88 and the initiator. Analysis via microwave analyser provides the solid content within minutes. However, the total conversion was confirmed later by determining the residual monomer by GC.

#### 2.1.3. Raman Spectroscopy

Raman spectroscopy without internal standard or calibration is a qualitative measuring method. Small changes in laser intensity or exposure time affect the intensity of the signal directly. Raman spectroscopy was therefore used solely for safety reasons in order to be able to detect a significant accumulation of the monomer at an early stage. The aim was, therefore, not to make quantitative statements, but merely to show that the monomer content remains constant over the entire process time.

Raman Spectroscopy was measured with a RamanRxn1-785 system from Kaiser Optical Systems (IO-1/2S-NIR probe, laser power 387 mW at the probe, Software: ICRaman). A new measurement was acquired every 32 s with an integration time of 30 s. The monomer content was tracked by monitoring the intensity of the peak at 1650 cm^−1^, which shows an overlap of the C=C bond of both monomer, vinyl acetate and Versa^®^10 [34]. The 12 mm probe was immersed into the initial charge at the opposite side of the PDW spectroscopy probe to avoid light interferences.

### 2.2. Inline Particle Size Measurement

PDW spectroscopy measurements were carried out inline, immersing the stainless-steel probe (2.5 cm diameter) directly into the reaction solution during the process. The probe was passed vertically into the vessel and maintained at equal distance between the stirrer and the wall of the reactor. The probe was far enough within the vessel for the optical fibres to protrude approximately 0.5 cm into the initial charge.

Three different wavelengths were used: 638 nm, 778 nm, and 855 nm. The device used was a Mini-PDW-spectrometer from the company PDW Analytics GmbH in collaboration with InnoFSPEC of the University of Potsdam. The measurements were processed with software based on Labview 2016. The refractive index and density of the copolymer were determined in a previous work and were implemented in the software [3].

### 2.3. Offline Determination of the Mean Particle Size

In addition to the inline measurement via PDW spectroscopy, the mean particle size was also determined by sedimentation analysis by means of DC and DLS. The refractive index and density of the copolymer were also implemented in the software of both measurement methods [3].

#### 2.3.1. Disc Centrifuge (DC)

Measuring the particle size by sedimentation analysis requires a gradient, which is why 0.2 mL of methanol was injected into the DC while at a halt. Then, the motor was started, and when reaching the maximum speed, 15 g of demineralised water was added steadily. One drop of the sample was diluted in 0.3 mL demineralised water and 0.1 mL methanol, and then 0.1 mL of the diluted sample was injected into the DC. The device used was a Disc Centrifuge DC24000 of the brand CPS. The given accuracy and repeatability lie at ±0.5% [35].

#### 2.3.2. Dynamic Light Scattering (DLS)

The mean particle size was also determined by dynamic light scattering using a Zetasizer Nano ZS from Malvern Instruments. One drop of the same sample, used previously, was diluted in 5 mL demineralised water and then measured in a polyethylene UV-cuvette. Each given particle size was obtained through a triple determination, each consisting of 18 measurements at 25 °C. A previous work provided a calibration of the device, which was used to correct the DLS measurements [3].

### 2.4. Determination of the Zeta Potential

The zeta potential was determined by a Zetasizer Ultra from Malvern Panalytical and using the ZS XPLORER software. The sample was diluted 200 times with MilliQ water (0.055 µS.cm^−1^ at 25 °C) and then measured in a disposable folded capillary cell (DTS1070) at 25 °C.

### 2.5. Determination of the Theoretical Particle Size

The determination of the theoretical particle size was based on the previously calculated number of particles. The number of particles (Np) was calculated based on the conversion and on the average particle size (dp) of the first sample with 10 wt% polymer content, measured by DC, as this was considered the reference measurement. The following equation was used:

$$\mathrm{Np}=\frac{3\times \;\chi \;\times {\;g}_{\mathrm{Monomer}}\;}{4\times \;\pi \;\times \;\rho \;\times {\left(\frac{\mathrm{dp}}{2}\right)}^{3}\;}\times 10^{21}$$

where χ is the monomer conversion determined by GC, g_Monomer_ is the amount of monomer dosed into the dispersion at the time of the sampling (g), ρ is the density of the copolymer (g.cm^−3^) [36], and dp is the particle size (nm) measured by DC.

It was assumed that the number of particles would stay constant throughout the reaction. The theoretical size of the other samples was then calculated accordingly.

## 3. Results and Discussion

### 3.1. Process Optimisation

The scale-up started with an optimisation of the process. As explained before in the previous chapter, all three reactors were kept as identical as possible in order to reduce possible cross-influences. In the past, Jacob et al. [3] published an emulsion polymerisation process that achieved a high polymer content of over 60 wt% in a 1 L glass reactor. However, the process revealed many complications, starting with the draining of the reactor and progressing to the forming of agglomeration as 40–50 wt% polymer content. This process worked at the time and formed a premise for the possibility of using PDW spectroscopy for industrial applications [20]. However, the process needed to be adjusted and optimised in order to establish whether upscaling could be accomplished without encountering safety issues due to heat generation. The new process, as described in the previous chapter, was therefore first carried out in an RC1 steel calorimeter to determine the heat generation during the process.

**Figure 2:**
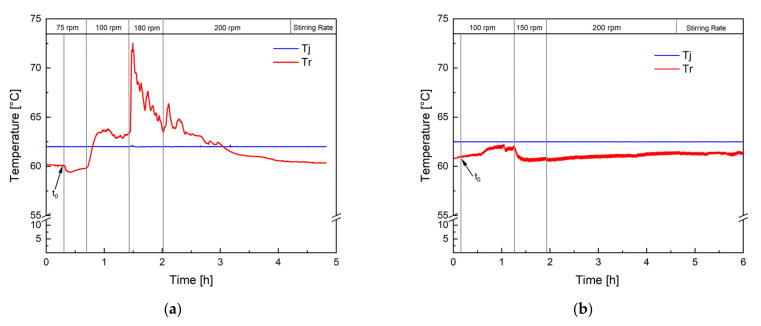
Temperature profile of two test reactions in a reaction calorimeter, one with insufficient mixing (**a**) and one with sufficient mixing (**b**). The reaction temperature (Tr) was monitored, and the temperature of the jacket (Tj) was kept constant at 62.5 °C. t_0_ indicates the initiation time.

Figure 2 shows the temperature profile of two polymerisation processes performed in an RC1 reaction calorimeter. The jacket temperature (Tj) was kept constant at 62.5 °C so that the reaction was performed in isoperibolic reaction control. Figure 2a clearly demonstrates the consequence of insufficient mixing: Tr increases by 10 °C after increasing the stirring rate from 75 to 100 rpm, indicating an accumulation of monomer now being consumed. Keeping the stirring rate constant throughout the entire process is therefore inadequate, and the stirring rate needs to be adjusted to the rising polymer content and viscosity of the dispersion. The temperature profile of the revised process (Figure 2b) showed that when choosing the right stirring rate, the constant jacket temperature supplies sufficient cooling to keep the reaction temperature in a constant, controllable range. Indeed, the calorimetric study showed a heat generation of 650 kJ and an increase in the reaction temperature (Tr) of a maximum of 2 °C at a constant jacket temperature. Considering a conversion of the monomer of 98%, which was confirmed via GC and microwave analysis, a heat generation of 920 kJ would have been expected (heat of polymerisation of vinyl acetate: −1036 J.g^−1^) [37]. This difference could be due to the fact that the dosing tanks were not heated, so the entire dosing volume was at room temperature when being transported into the reactor. Considering that over 60% of the final charge was dosed during the process, this leads to a significant cooling, which is not considered by the calorimetric measurement and heat-generation calculation. However, this additional cooling facilitated the control over the reaction temperature, even more at greater reaction volumes, such as 100 L, and was therefore maintained. Moreover, 4.18 kJ is required to heat up 1 kg of water by 1 °C. Considering that Tr increased by a maximum of 2 °C, good control over the polymerisation heat can be concluded, and no risk of a dangerous increase in the reaction temperature was to be expected during the scale-up process. The calorimetric study, therefore, showed that the optimised process could be carried out safely; even in the case of a temporary stirring or cooling failure, only the dosing needs to be interrupted until the problem is solved.

Additional inline monitoring of the monomer via Raman spectroscopy offered the possibility of identifying resulting problems in the process, enabling a quick intervention to avoid problematic heat generation (cf. exemplary Raman trend, see Supplementary Materials Figure S1).

As a result of the preliminary test runs, it was possible to safely adjust the process to the three reactor sizes of 1 L, 10 L, and 100 L, as described before in Table 2 and Table 4.

### 3.2. Scale-Up Process and Safety Precautions

**Figure 3:**
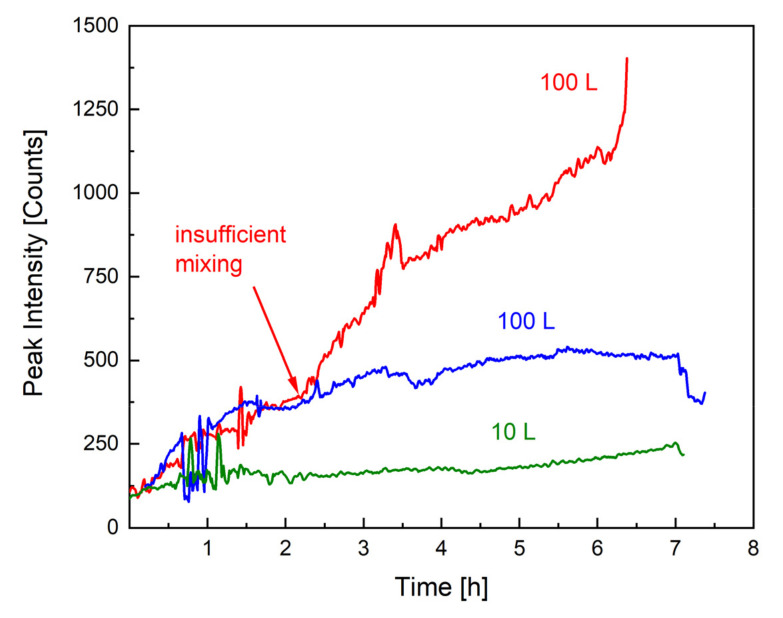
Monomer concentration over time, tracked by Raman spectroscopy (1646 cm^−1^). The green and blue trends show the course of the Raman peak throughout an emulsion polymerisation in, respectively, 10 and 100 L. The red line describes the monomer accumulation when mixing is insufficient.

As mentioned previously, the most important safety precaution in the upscaling of emulsion polymerisations is to avoid an uncontrollable accumulation of monomer in the dispersion, which could lead to a considerable heat generation. The monomer concentration can be influenced by the dosing rate but also by the reaction rate and mixing properties. For example, the dosing rate must not exceed the reaction rate, and the dispersion must be kept homogenous by avoiding insufficient mixing, as demonstrated in Figure 2. Figure 3 illustrates the effect of insufficient mixing on the monomer accumulation (monitored by Raman spectroscopy). Without an internal standard or calibration, Raman spectroscopy only provides qualitative information on monomer content, and the difference in the peak intensity between the 10 L (green) and 100 L (blue) scale can be related to a different exposure time as well as total monomer amount. GC measurements of all samples showed a conversion of at least 98% at all times. The remaining 2% monomer forms a smaller amount at a 10 L scale than at a 100 L scale, which could further explain the higher intensity of the signal. However, the Raman analysis indicates that the monomer concentration present in the dispersion remains constant across scales and throughout the process (both intensities in blue and green), whereby the intensity of the monomer peak of the second 100 L trend (red) clearly indicates that the dosed monomer cannot be fully consumed. The only difference between the reaction in blue and the one in red is the stirring motor and rate, clearly demonstrating how important mixing is, not only for safety reasons but also for the desired product properties (cf. next section).

### 3.3. Comparison of Particle Size Measuring Methods

**Figure 4:**
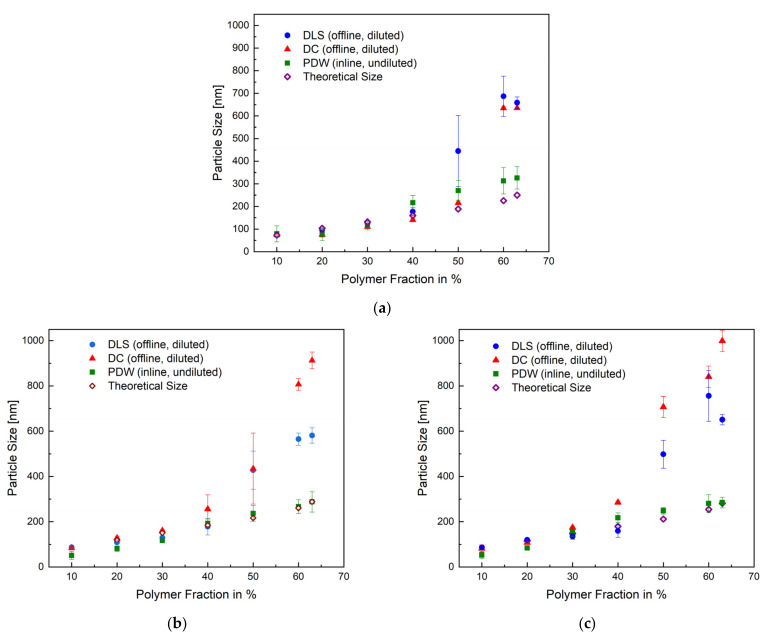
Overview of the measured mean particles size of all three measurements methods and the calculated theoretical size as a function of the polymer fraction with the respective standard deviation for the emulsion polymerisation in a 1 L (**a**), 10 L (**b**), and 100 L (**c**) reactor.

After achieving a reproducible, thermally safe process, the particle sizes obtained by each measurement method were represented as a function of the polymer content of the respective sample in Figure 4. In addition to the offline (DLS, DC) and inline (PDW) measurement methods, the theoretically predicted particle size was calculated and compared to all measuring methods. The overview in Figure 4 shows the mean particle size of each method for the emulsion polymerisation process in a 1 L, 10 L, and 100 L reactor. All methods correlate up until 40 wt%. At 50–63 wt%, the measurements by DLS and DC show a deviation of, respectively, 100% and 200% in relation to the PDW measurement and calculated theoretical size. The PDW measurements agree with the theoretically calculated particle size. The deviation observed between the offline and inline methods could suggest that cooling down the process leads to an alteration of the product—for example, post-process agglomeration. It is possible to assume that increasing the solid content also increases the probability of the coalescence of particles, besides the fact that polyvinyl alcohol has a tendency to lead to “clathrate-like” structure promotion. [38,39] An additional cooling of the sample in combination with a lack of stirring could intensify this even more. The exact values for all measured particle sizes and their respective standard deviation are summarised in Tables S1–S3 in the Supplementary.

**Figure 5:**
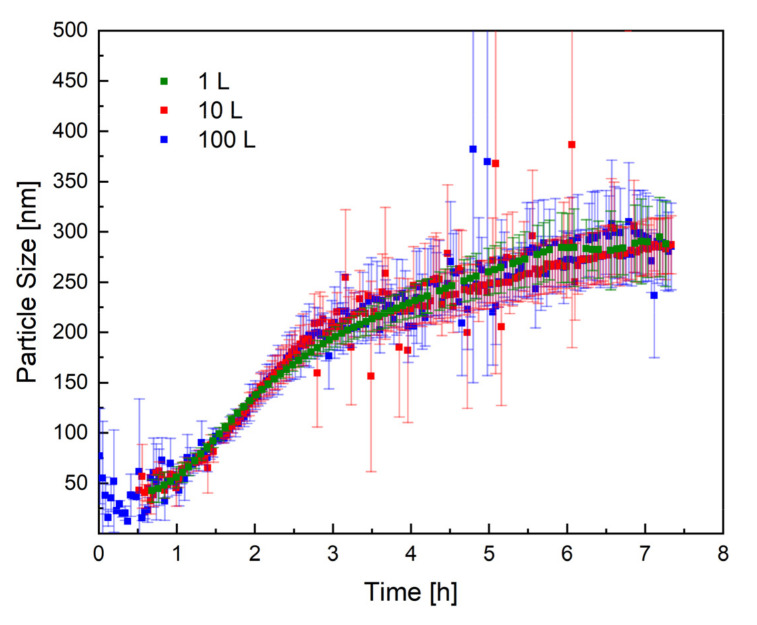
Representation of the particle size as a function of the reaction time in all three reactor sizes: 1 L (green), 10 L (red), and 100 L (blue). The standard deviation of each measurement is shown in the respective colour.

Figure 5 shows the mean trend of the particle size, measured by PDW spectroscopy, in all three reactor sizes. The trend of the particle size in all three reactor sizes (1 L, 10 L, and 100 L) is practically identical, which proves that the process has very good reproducibility, even in different reactor sizes. The scale-up of the redox initiated emulsion polymerisation of vinyl acetate and Versa^®^10 was therefore successful regarding reproducible particle growth.

### 3.4. Redispersion of the Product

**Figure 6:**
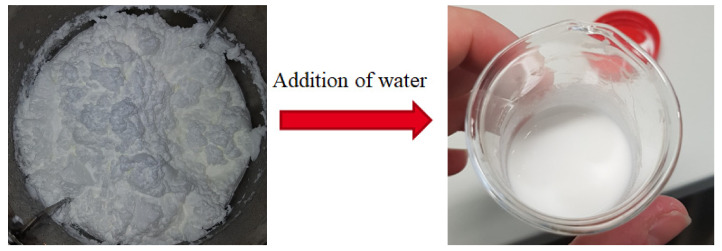
Successful redispersion of the product by adding water.

Increasing the polymer content to over 60 wt% while keeping the process manageable and the product stable can be very challenging, especially with an already known tendency for agglomeration. With an increasing polymer content, the dispersion grows thicker and is much less fluid than at 20–40 wt%. With a decreasing fluidity of the dispersion, the probability of gelation increases, and the ratio of protective colloid to polymer decreases. This can lead to less stability and increase the risk of agglomeration. Therefore, once the final product with 63–67 wt% was obtained and a sample was taken for further analysis, water was added to the dispersion to lower the polymer content. Adding water to a stable dispersion will lead to an increase in fluidity without altering its properties and appearance. When adding water to an instable dispersion, lumps will occur, and the additional water will not diffuse thoroughly into the dispersion. Figure 6 shows the obtained final product on the left side, with a polymer content of 67 wt%. The product did not seem to be smooth anymore and was rather creamy. After adding some water to the sample, the dispersion became fluid and smooth again (cf. Figure 6, right), which hints at a stable product.

Particle size measurement via DLS of the dispersed sample showed no change compared to before, and the measured zeta potential of −12 mV was comparable to a commercially available Vinnapas^®^ dispersion of polyvinyl acetate. Considering the commonly known tendency of polyvinyl acetate to agglomerate, this can be considered to be a stable dispersion.

## 4. Conclusions

The emulsion polymerisation of vinyl acetate and Versa^®^10 is known to tend to agglomerate at high solid contents. For this polymerisation system, a scale-up process up to a polymer content of 67 wt% was successfully and reproducibly achieved in a scale-up process from 1 L, through 10 L, and up to 100 L via a redox-initiated starved–fed semi-batch process. Safety precautions, such as inline monitoring of monomer content via Raman spectroscopy (qualitative monomer accumulation) and calorimetric studies, were implemented to ensure the possibility of identifying any resulting risk and simultaneously enable a quick intervention to avoid accidents. In addition, the recipe was optimised to ensure a stable and reproducible process, with an increase in the reaction temperature to a maximum of 2 °C and no lump formation.

PDW spectroscopy proved to be a reliable and precise measuring method for the inline monitoring of particle size, even at a higher scale and with polymer contents up to 67 wt%, showing a clear advantage in comparison to DLS or DC. All determined mean particle diameters concurred with the DLS and sedimentation analysis measurements up to a polymer content of 40 wt%. PDW spectroscopy continues to concur with the calculated theoretical particle size at even higher polymer contents up to 67 wt%, while DLS and DC showed difficulties, which could be due to post-process alteration of the dispersion.

PDW spectroscopy also displayed the reproducibility of the process in all reactor sizes and even offered reliable information on the mean particle size of creamy and slightly lumpy emulsions. The measurement method has therefore proven to be promising not only on a laboratory scale but also on an industrial scale. However, further investigations should be carried out in order to emphasise the possibility of obtaining even more information by analysing the scattering coefficient also measured by PDW spectroscopy. 

Future work could also investigate whether the dispersion is subject to post-process or cooling agglomeration and whether this is a reversible process.

## Figures and Tables

**Table 1:** Overview of the measurements of the experimental set-up of all three reactor sizes, represented in Figure 1.

(In cm)	1 L	10 L	100 L
H	20.5	27	60
H1	3	6	12
h	1	1.5	5
D	12	24	48
d	10.4	20.8	41.6

**Table 2:** Overview of the stirring rates during the emulsion polymerisation in all three reactor sizes.

(In rpm)	1 L	10 L	100 L
Stirrer Rate (beginning)	300	75	60
Stirrer Rate (as of 30 wt%)	400	120	60
Stirrer Rate (as of 45 wt%)	500	240	90

**Table 3:** Overview of the Reynolds number for the different reactor sizes.

(.10^3^)	1 L	10 L	100 L
Reynolds number (beginning)	40	40	120
Reynolds number (as of 30 wt%)	50	60	120
Reynolds number (as of 45 wt%)	75	120	180

**Table 4:** Overview of the initial charge and feeding rates.

	1 L	1.8 L	10 L	100 L
Dem. Water	244 g	488	2440 g	27,500 g
Mowiol 4–88	12.3 g	24.6	123 g	1383.5 g
Monomer Feed	1.2 mL.min^−1^	2.4 mL.min^−1^	12 mL.min^−1^	120 mL.min^−1^
Redox Feed	0.08 mL.min^−1^	0.16 mL.min^−1^	0.84 mL.min^−1^	8.4 mL.min^−1^

**Table 5:** Composition of the redox system: concentration of the redox feed and overall ratio of oxidising and reducing agent to the catalyst.

	Concentration of Feed	Molar Ratio Redox System
	g.L^−1^	
AsAc	60	1
tBHP	57	1.3
Fe-cat.	-	0.01

## Data Availability

The authors confirm that the data supporting the findings of this study are available within the article and its Supplementary materials. Further data is available upon request from the corresponding author.

## Supplementary Materials

The following supporting information can be downloaded at: https://www.mdpi.com/article/10.3390/polym14081574/s1, Figure S1: Exemplary representation of the inline monitoring of the monomer accumulation via Raman spectroscopy; Table S1: Overview of the measured particle size and respective standard deviation by PDW spectroscopy, DC, DLS and the calculated theoretical size for the emulsion polymerisation in a 1 L reactor; Table S2: Overview of the measured particle size and respective standard deviation by PDW spectroscopy, DC, DLS and the calculated theoretical size for the emulsion polymerization in a 10 L reactor; Table S3: Overview of the measured particle size and respective standard deviation by PDW spectroscopy, DC, DLS and the calculated theoretical size for the emulsion polymerization in a 100 L reactor.
