# Supplementary material for: Scale-up of Emulsion Polymerisation up to 100 L and with a Polymer Content of up to 67 wt%, Monitored by Photon Density Wave Spectroscopy
Source: Polymers (Basel). 2022 Apr 12;14(8):1574. doi: 10.3390/polym14081574 (PMC9028448; doi:10.3390/polym14081574)
Supplement: Supplementary file 1 [file polymers-14-01574-s001.zip › polymers-1600941-supplementary.pdf]

polymers

MDPI

Supplementary Materials

Supplementary Materials: Scale-Up of Emulsion Polymerisation Up to 100 L and with a Polymer Content of Up to 67 wt%, Monitored by Photon Density Wave Spectroscopy

Laurence Isabelle Jacob and Werner Pauer

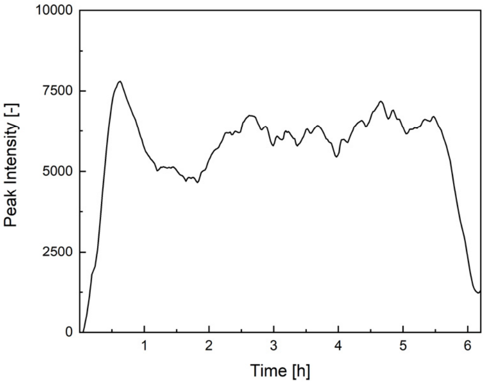

Figure S1. Exemplary representation of the inline monitoring of the monomer accumulation via Raman spectroscopy.

Table S1. Overview of the measured particle size and respective standard deviation by PDW spectroscopy, DC, DLS and the calculated theoretical size for the emulsion polymerisation in a 1 L reactor.

Polymer Fraction	Theoretical Size	PDW	DC (Number Mean)	DLS (Number Mean)
wt%	nm	nm	nm	nm
10		79 ± 35	73 ± 3	74 ± 9
20	104	75 ± 27	75 ± 7	96 ± 9
30	132	115 ± 18	111 ± 3	125 ± 11
40	159	217 ± 32	141 ± 4	177 ± 17
50	189	270 ± 44	216 ± 4	445 ± 157
60	226	313 ± 58	635 ± 6	687 ± 89
63	250	326 ± 49	635 ± 6	659 ± 26

Table S2. Overview of the measured particle size and respective standard deviation by PDW spectroscopy, DC, DLS and the calculated theoretical size for the emulsion polymerization in a 10 L reactor.

Polymer Fraction	Theoretical Size	PDW	DC (Number Mean)	DLS (Number Mean)
wt%	nm	nm	Nm	nm
10		51 ± 18	84 ± 9	87 ± 5
20	119	81 ± 12	128 ± 9	109 ± 1
30	151	118 ± 11	161 ± 3	130 ± 5
40	184	193 ± 19	256 ± 63	178 ± 36
50	217	237 ± 35	435 ± 157	428 ± 84
60	260	267 ± 30	807 ± 26	565 ± 27
63	288	288 ± 45	913 ± 37	581 ± 34

Table S3. Overview of the measured particle size and respective standard deviation by PDW spectroscopy, DC, DLS and the calculated theoretical size for the emulsion polymerization in a 100 L reactor.

Polymer Fraction	Theoretical Size	PDW	DC (Number Mean)	DLS (Number Mean)
wt%	nm	nm	Nm	nm
10		53 ± 16	82 ± 16	87 ± 6
20	116	83 ± 10	108 ± 5	120 ± 9
30	148	156 ± 20	174 ± 4	135 ± 12
40	179	218 ± 22	285 ± 7	159 ± 28
50	212	248 ± 14	707 ± 47	498 ± 61
60	254	280 ± 39	841 ± 45	756 ± 112
63	281	284 ± 24	999 ± 50	651 ± 23
